# The Application of Machine Learning ICA-VMD in an Intelligent Diagnosis System in a Low SNR Environment

**DOI:** 10.3390/s21248344

**Published:** 2021-12-14

**Authors:** Shih-Lin Lin

**Affiliations:** Graduate Institute of Vehicle Engineering, National Changhua University of Education, No. 1, Jin-De Road, Changhua City 50007, Taiwan; lin040@cc.ncue.edu.tw

**Keywords:** machine learning, independent component analysis, variational mode decomposition, low SNR, fault diagnosis

## Abstract

This paper proposes a new method called independent component analysis–variational mode decomposition (ICA-VMD), which combines ICA and VMD. The purpose is to study the application of ICA-VMD in low signal-to-noise ratio (SNR) signal processing and data analysis. ICA is a very important method in the field of machine learning. It is an unsupervised learning algorithm that can dig out the independent factors hidden in the observation signal. The VMD method estimates each signal component by solving the frequency domain variational optimization problem, and it is very suitable for mechanical fault diagnosis. The advantage of ICA-VMD is that it requires two sensory cues to distinguish the original source from the unwanted noise. In the three cases studied here, the original source was first contaminated by white Gaussian noise. The three cases in this study are under different SNR conditions. The SNR in the first case is –6.46 dB, the SNR in the second case is –21.3728, and the SNR in the third case is –46.8177. The simulation results show that the ICA-VMD method can effectively recover the original source from the contaminated data. It is hoped that, in the future, there will be new discoveries and advances in science and technology to solve the noise interference problem through this method.

## 1. Introduction

Sensor measurement obtains valuable data, which have been widely used in many applied sciences and engineering fields. However, analyzing these measurement data is indispensable and extremely challenging. Analyzing data is essential for research, because, through this process, evidence of research findings can be established. Data analysis can be divided into two types. One type of data can be directly interpreted, which means that the value of the number can be mapped to the system and defined. The other type is that the sensor measurement is interfered by the external environment and cannot be directly explained. It is necessary to understand the original features hidden in the data through data analysis or signal processing. This is difficult, but is an important process of scientific progress and new discoveries.

For example, in the sensor measurement system, the characteristic information of the sensing data is submerged in noise. The manner in which to effectively extract this characteristic information has always been an important topic in the research of equipment condition monitoring and fault diagnosis. Wavelet analysis [1,2,3] and empirical mode decomposition (EMD) [4,5,6] are currently commonly used fault feature extraction methods, but wavelet analysis produces energy leakage when processing signals due to the limited length of the basis function. In addition, the choice of wavelet basis function is also a difficult problem. For a certain signal, the principles and criteria that should be used to select wavelet basis are still to be studied in theory and practice. EMD is not supported by complete mathematical theory as is wavelet transform. It is a modal decomposition based on “experience,” which affects the further development and application of empirical mode decomposition theory. However, EMD also has shortcomings, that is, the problem of mode mixing effect and end effect. Dragomiretskiy et al. [7] proposed a variational mode decomposition (VMD) method. This new signal estimation method is different from the recursive mode of EMD and local mean decomposition. It is a non-recursive signal decomposition method that presupposes that each modal component has a center frequency to determine each component. The VMD method has a theoretical basis. The end and mode mixing effects of the VMD method are much smaller than those of EMD and LMD (local mean decomposition) [8]. The biggest feature of the LMD algorithm lies in its ability to adaptively decompose a signal according to the characteristics of the signal itself to produce a product function (PF) component with real physical meaning. From this, a time–frequency distribution that can clearly and accurately reflect the distribution of signal energy on various scales in space is obtained, which is conducive to more detailed analysis of signal characteristics. There are many documents on the application of VMD in different fields, and it has a lot of contributions [9,10,11,12].

In the fields of neural networks, statistical analysis, signal processing, etc., a common concern is how to find an appropriate representation of the source data with the help of a certain appropriate transformation. Linear transformation techniques such as the well-known principal component analysis (PCA) [13], factor analysis (FA) [14,15], and projection tracking (PP) [16] have been widely used. Blind source separation is a method of estimating channel parameters and restoring the source signal based on the statistical characteristics of the input observation signal when the source signal and transmission channel parameters are unknown. Blind source separation has been widely used in image processing, speech processing, biomedicine, seismic detection, and other fields, and has become a research hotspot in the field of signal processing. Independent component analysis (ICA) is a feature extraction method based on sample high-order statistical information developed in recent years. It belongs to an unsupervised feature extraction method. The basic idea is to first assume that the sample set is composed of a set of mutually independent basis vectors and the corresponding mixing matrix, and then use the corresponding algorithm to find the unmixing matrix (the inverse matrix of the mixing matrix).

Bell and Sejnowski [17] proposed a single-layer feedforward neural network algorithm. The Infomax algorithm effectively separates the linear mixed signals of multiple super-Gaussian source signals. Lee and Girolami [18], on the basis of retaining Bell’s basic algorithm system, combined the natural gradient and maximum likelihood estimation method proposed by Gaussian mixed signal. Amari, Cichocki, and Yang proposed [19] an efficient minimization of mutual information. The α learning rule maximizes negative entropy. Cardoso and Souloumiac [20] found that mutual information can be decomposed into two parts through linear transformation. One part represents the decorrelation of the components, and the other represents their non-Gaussianness. In addition, the relationship between non-Gaussian measures is also discussed. Although the most direct way to measure the independence between independent components is to use the probability density function (PDF), it is more difficult and cumbersome to directly obtain the PDF, and it is necessary to indirectly estimate the PDF or related quantities. Blanco and Zazo [21] proposed a number of new measures that describe the statistical distance between two distributions based not on PDF, but on the cumulant density function, and gave their application in constructing the ICA objective function. Learned-Miller and Fisher [22] avoided estimating PDF; instead, they directly used entropy as a measurement, and use the micro-brain estimation method of continuous random variables to propose a new ICA solution based on entropy-based distance estimation. Eriksson and Koivunen [23] proposed a number and a second characteristic function based on the characteristics of random variables. The new ICA solution using the Jacobi-type optimization algorithm obtains a unique definition based on the factorization of the joint characteristic function. Boscolo et al. [24] used non-parametric estimation of PDF to solve the ICA problem, and at the same time estimated the unknown PDF and separation matrix of the source signal. Although ICA has many advantages in data analysis, it also has great disadvantages. The number of sensors should be greater than or equal to the number of sources. It has been found that with the development of over- or under-complete representations [25,26], not all raw data can be separated by a small number of sensors. There are many documents on the application of ICA in different fields, and it has many contributions [27,28,29,30,31,32].

Lin [33,34] proposed the intelligent fault diagnosis of bearings based on the classification and prediction of VMD analysis signals and deep learning. But the study is in the absence of noise interference. Hua et al. [35] proposed a laser radar echo signal denoising method based on parameter optimal variational mode decomposition (VMD) combined with Hausdorff distance (HD) and wavelet transform (WT), but the SNR of this study is 4 dB. Wang et al. [36] proposed using VMD decomposition to adaptively decompose the original signal into multiple IMF. It uses an independent component analysis algorithm based on kurtosis maximization to achieve the best estimation of transient electromagnetic signals, but the SNR of this study is −8 dB. Wang et al. [37] proposed the blind source extraction of rail crack acoustic emission signals based on ensemble empirical mode decomposition and constrained independent component analysis. Li et al. [38] proposed multi-source feature extraction of rolling bearing compression measurement signals based on independent component analysis. Imajo et al. [39] proposed to separate signal and noise from satellite magnetic field data through independent component analysis. However, ICA has a big limitation. Several sensors can only analyze a few original signals, and two sensors can only analyze two source signals. Because the researcher cannot know how many source signals are in use in the system, and the number of sensors is limited. It is important to emphasize that the order of the two methods of ICA and VMD is very important.

Take mechanical fault diagnosis, as an example, to extract the fault characteristics of the mechanical vibration signal for maintenance. The working conditions of mechanical equipment during normal operation are complex and changeable. The generated vibration signal is easily affected by strong background noise, acquisition equipment, and acquisition signal transmission equipment, and it is not easy to observe fault information. When using VMD directly to decompose the collected mechanical fault vibration signals, if the results obtained are affected by noise, the fault signals of certain components cannot be found. Therefore, although the traditional VMD noise reduction method can reduce some noise interference, it still has its limitations. It cannot be analyzed in very noisy environments with low SNR. Therefore, in order to improve the effective signal-to-noise ratio of the collected original vibration signal and highlight the fault characteristic information, reducing the noise of the original signal is an indispensable process. The contribution of this research is the study ICA for many years. It was found that receiving or measuring signals through more than two sensors can solve the low SNR in the environment of noise interference. If the original signal is disturbed by noise, the feature extraction method of ICA’s high-order statistical information can obtain the result of more than two components. One component is noise, and the other component is the original signal, which realizes the work of noise filtering. Through the preprocessing of ICA, VDM can use the original feature extraction characteristics. In the three cases studied herein, the original source was first contaminated by white Gaussian noise. The three cases in this study are in different SNR scenarios. The SNR in the first case is –6.46 dB, the SNR in the second case is –21.3728, and the SNR in the third case is –46.8177. In this study, ICA-VMD uses 2 sensors to separate five source signals in a low SNR environment with high noise. Using ICA or VMD alone cannot achieve such results. The simulation results show that the ICA-VMD method can effectively recover the original source from the noise interference data. VMD decomposition decomposes the signal into multiple components, thereby more effectively extracting the characteristics of each frequency band in the signal.

The following outline describes the structure of the entire paper. Section 1 introduces research motivation, research and importance, related references and explanations of research originality. In Section 2, the research methodology introduces the combination and flow chart of ICA and VMD. According to the research results in Section 3, ICA-VMD was used in three cases. In each case, firstly, only VMD was used, and then ICA-VMD was used. The conclusions in Section 4 explain the research results, contributions and research limitations. ICA-VMD can be applied to other areas to solve the problem of noise interference.

## 2. Research Methodology

ICA is an unsupervised learning algorithm that is very important in the field of machine learning. It is assumed that the components of the source signal $S\left(t\right)={\left[s_{1}\left(t\right),s_{2}\left(t\right),\cdots ,s_{N}\left(t\right)\right]}^{T}$ are statistically independent, and that they are linearly mixed through the system to obtain the observation signal ***X***(*t*). The relationship between the observed signal and the source signal can be expressed by the following equation:

(1) $$x_{i}\left(t\right)=\overset{N}{\underset{j=1}{\sum }}a_{ij}s_{j}\left(t\right),\;\;\;\;\;i=1,2,\ldots ,M$$

or expressed in matrix form as:

(2) X(t)=AS(t)

where ***A*** is an *M* × *N* order matrix.

The purpose of independent component analysis is to find a linear transformation matrix **W** [18] to make the signals, after transforming ***X***(t), as statistically independent as possible, and to use these independent signals as an estimate of the source signal.

Let $Y\left(t\right)={\left[y_{1}\left(t\right),y_{2}\left(t\right),\cdots ,y_{N}\left(t\right)\right]}^{T}$ be the output after unmixing; then:

(3) Y(t)=WX(t)

where **W** is an *N* × *M* order matrix, also called an unmixing matrix.

It is difficult to directly solve the mixing matrix ***A***. Usually, the solution mixing matrix is divided into whitening and orthogonal transformation. The ultimate goal of ICA is to decompose mutually independent signals. The signal independence is stricter than uncorrelated, and whitening is a property between independent and unrelated. The whitening of observation data can be obtained by linear transformation, namely:

(4) Z(t)=VX(t)

where ***Z***(*t*) is the whitening data.

The most commonly used whitening method is to perform eigenvalue decomposition on the covariance matrix of the observation data [18], and the steps are as follows.

(1) Perform eigenvalue decomposition on the covariance matrix to achieve eigenvalue decomposition to obtain eigenvalues and eigenvectors:

(5) $$E\left\{XX^{T}\right\}=EDE^{T}$$

where ***E*** is the eigenvector, and ***D*** is the symmetric matrix composed of eigenvalues.

(2) Calculate the whitening matrix, which can be achieved via the following equation:

(6) $$V=ED^{-1/2}E$$

From Equations (1) and (4):

(7) $$Z\left(t\right)=VAS\left(t\right)=\tilde{A}S\left(t\right)$$

Therefore:

(8) $$E\left\{Z\left(t\right)Z{\left(t\right)}^{T}\right\}=\tilde{A}E\left\{S\left(t\right)S{\left(t\right)}^{T}\right\}{\tilde{A}}^{T}=\tilde{A}{\tilde{A}}^{T}$$

Moreover, $\left\{Z\left(t\right)Z{\left(t\right)}^{T}\right\}=I$, so $\tilde{A}{\tilde{A}}^{T}=I$. In this way, the problem of solving matrix A is transformed into solving matrix $\tilde{A}$. There are only n(n−1)/2 free variables, so the signal separation problem of ICA after whitening is simplified.

The method of joint approximation diagonalization of feature matrix is described here. Let ***M*** be any *N* × *N*-order matrix; the *ij* elements ***Q****_z_* (***M***)*_ij_* of the four-dimensional cumulant matrix ***Q****_z_* (***M***) of ***Z*** can be expressed as [20]:

(9) $$Q_{z}{\left(M\right)}_{ij}=\overset{N}{\underset{k=1}{\sum }}\overset{N}{\underset{l=1}{\sum }}K_{ijkl}\cdot m_{kl},\;\;\;\;i,j=1,2,\cdots ,N\;$$

where $K_{ijkl}\left(z\right)$ is the four-dimensional cumulant of the four components *i, j, k*, and *l* in the loss z; ***Q****_z_*(***M***) is also an *N* × *M* order matrix; $m_{kl}$ is the *k, l* element of the matrix ***M***. ***Q****_z_* (***M***) can be expressed as [13] with its eigenvalue and eigenmatrix.

(10) $$Q_{z}\left(M\right)=\tilde{A}\Lambda \left(M\right){\tilde{A}}^{T}$$

(11) $$\Lambda \left(M\right)={\tilde{A}}^{T}Q_{z}\left(M\right)\tilde{A}=diag\left[kurt\left(s_{1}\right){\tilde{a}}_{1}M{\tilde{a}}_{1}^{T},\cdots ,kurt\left(s_{N}\right){\tilde{a}}_{N}M{\tilde{a}}_{N}^{T}\right]\;$$

This equation shows that the quadratic processing of matrix $\tilde{A}$ on ***Q****_z_*(***M***) can obtain the orthogonal matrix $\tilde{A}$, and the calculation of the diagonal matrix Λ(***M***) can also calculate Λ(***M***). The method is as follows:

(1) Whitening: According to Equation (7), whiten the observation data.
(2) Select matrix ***M***, and estimate the cumulant matrix ***Q****_z_*(***M***) using Equation (9) according to the observation data.
(3) Calculate the mixed-whitening matrix $\tilde{A}$: According to Equation (10), the matrix that can make ***Q_z_***(***M***) diagonal is $\tilde{A}$.
(4) Estimate the separation matrix ***W*** and separate the observation signal:
  $$\hat{A}=V^{-1}\tilde{A},W={\hat{A}}^{-1}={\tilde{A}}^{-1}V,Y=WX={\tilde{A}}^{-1}VX$$

In actual use, it is found that the result obtained by taking only one ***M*** array and following the above steps is not ideal. In order to get better results, take *P* matrices ***M*** = [*M_1_,M_2_,…,M_p_*], and calculate ***Q****_z_*(***M***_i_)(*i* = 1,2,…,*P*) for each ***M****_i_*, and then look for matrix $\tilde{A}$. The required period can make each ***Q****_z_*(***M****_i_*) as diagonal as possible. In order to give a quantitative measure of the degree of non-diagonalization of Λ(***M****_i_*) = ${\tilde{A}}^{T}$***Q****_z_*(***M****_i_*)$\tilde{A}$, the sum of the squares of the non-diagonal elements in each Λ(***M****_i_*) is used as the objective function:

(12) $$D_{M}\left(V\right)=\underset{M_{i}\in M}{\sum }off\left[\Lambda \left(M_{i}\right)\right]=\underset{M_{i}\in M}{\sum }off\left[{\tilde{A}}^{T}Q_{z}(M_{i}\right)\tilde{A}]\;\;\;\;$$

Therefore, the actual ICA procedure is as follows:

(1) Whitening: Perform whitening according to the observation data of Equation (7).
(2) For all the matrices ***M****_i_*, estimate the cumulant matrix ***Q****_z_*(***M****_i_*) using Equation (9) according to the observation data.
(3) Through optimized calculation of $\tilde{A}$, make all ***Q****_z_*(***M****_i_*) joint approximate diagonalization, even if the criterion makes (12) as small as possible.
(4) Estimate the separation matrix ***W*** according to the method in step (4) above and separate the observed signal.

Although joint approximation diagonalization of eigenmatrices (JADE) is not commonly used, the performance of this type of JADE is particularly good after studying various ICA theories. Other ICA theories tend to fail to restore the original signal. For related theories of JADE, please refer to the literature [20].

VMD is an adaptive [7], completely non-recursive modal variation and signal processing method. The signal adaptive decomposition is realized by searching for the optimal solution of the constrained variational model. First, intrinsic mode functions are defined as amplitude-modulated–frequency-modulated signals, and the expression is

(13) $$u_{k}\left(t\right)=A_{k}\left(t\right)\cos\left(\phi_{k}\left(t\right)\right)$$

In the above equation, $A_{k}\left(t\right)$ is taken as the instantaneous amplitude of $u_{k}\left(t\right)$, and $A_{k}\left(t\right)$ ≥ 0; $\phi_{k}\left(t\right)$ is used as the instant phase of $u_{k}\left(t\right)$, and $\phi_{k}\left(t\right)$ obtains the first-order differential of t to obtain the instantaneous frequency of $u_{k}\left(t\right)$:

(14) $$w_{k}\left(t\right)=\frac{\phi_{k}\left(t\right)}{dt},w_{k}\left(t\right)\geq 0.$$

The goal of estimating the frequency domain width of each IMF component by creating a variational pattern:

Perform Hilbert transformation on the modal function $u_{k}\left(t\right)$. Get its analysis input:

$$\left(\delta \left(t\right)+\frac{j}{\pi t}\right)\cdot u_{k}\left(t\right)$$

where

(15) $$\delta \left(t\right)=\{\begin{array}{c} 0\;\;t\neq 0\\ \infty \;t=0 \end{array},\;\int_{-\infty }^{+\infty }\delta \left(t\right)dt=1.$$

Add an estimated center frequency $e^{-j\omega_{k}t}$ to the analytical signal of each mode, so that the frequency spectrum of each mode can be modulated to the corresponding base band

(16) $$\left[\left(\delta \left(t\right)+\frac{j}{\pi t}\right)\cdot u_{k}\left(t\right)\right]e^{-j\omega_{k}t}$$

Derive the norm gradient square L2 in Equation (16), estimate the width of the $u_{k}\left(t\right)$ modal function, the initial variational constraint problem. Assuming that each mode $u_{k}\left(t\right)$ has a center frequency and a limited bandwidth, the constraint condition is that the sum of each mode is equal to the input signal f, and the sum of the estimated bandwidth of each mode is the smallest, and its constrained variational model is as follows [7]:

$$\underset{\left\{u_{k}\right\}\left\{\omega_{k}\right\}}{\min}\left\{\underset{k}{\sum }||\partial_{t}\left[\left(\delta \left(t\right)+\frac{j}{\pi t}\right)*u_{k}\left(t\right)\right]e^{-j\omega_{k}t}|{|}_{2}^{2}\right\}$$

(17) $$s.t.\;\;\;\;\underset{k}{\sum }u_{k}=f$$

In the equation, $\left\{u_{k}\right\}\;:=\left\{u_{1},\ldots ,u_{K}\right\}$ represents the *K* finite bandwidth IMF components obtained by decomposition, and $\left\{\omega_{k}\right\}\;:=\left\{\omega_{1},\ldots ,\omega_{K}\right\}$ represents the frequency center of each component $\Sigma_{k}\;:=\Sigma_{k-1}^{K}$.

The extended Lagrange is introduced to turn the constrained variational problem into a non-constrained variational problem, and the optimal solution of Equation (2) is obtained, and its expression is:

(18) $$\begin{array}{cc} L\left(\left\{u_{k}\right\},\left\{\omega_{k}\right\},\lambda \right) & :=\alpha \underset{k}{\sum }||\begin{array}{c} \partial_{t}\left[\left(\delta \left(t\right)+\frac{j}{\pi t}\right)*u_{k}\left(t\right)\right]e^{-j\omega_{k}t}|{|}_{2}^{2} \end{array}\\ & +||f\left(t\right)-\underset{k}{\sum }u_{k}\;\left(t\right)|{|}_{2}^{2}+\langle \lambda \left(t\right),f\left(t\right)-\underset{k}{\sum }u_{k}\left(t\right)\rangle . \end{array}$$

In the equation, α is the penalty parameter to ensure the accuracy of signal reconstruction, and λ is the Lagrange multiplier, which keeps the constraint conditions strict. While, δ(t) is the average pulse function; $\partial_{t}$ is the first-order partial derivative of the functional with respect to time *t*; *j* is the imaginary unit;∙ ∗ is the convolution symbol.

In order to solve the above variational problem, the alternate direction method of multipliers (ADMM) is used to alternately update $u_{k}^{n+1},\;\omega_{k}^{n+1}$ and $\lambda^{n+1}$, and to find the “saddle point” of Equation (3), where the expression of $u_{k}^{n+1}$ is:

(19) $$u_{k}^{n+1}=\arg\underset{u_{k}\epsilon X}{\min}\left\{\alpha ||\partial_{t}\left[\left(\delta \left(t\right)+\frac{j}{\pi t}\right)*u_{k}\left(t\right)\right]e^{-j\omega_{k}t}|{|}_{2}^{2}+||f\left(t\right)-\underset{i}{\sum }u_{i}\left(t\right)+\frac{\lambda \left(t\right)}{2}|{|}_{2}^{2}\right\},\;\;$$

In the equation, $\omega_{k}$ is equivalent to $\omega_{k}^{n+1},$ $\sum_{i}u_{i}\left(t\right)$, which is equivalent to $\sum_{i\neq k}u_{i}{\left(t\;\right)}^{n+1}$.

Based on Parseval/Plancherel Fourier equidistant transform, Equation (19) is transformed into the frequency domain, and the frequency domain update of each mode is obtained. Then the value of the center frequency is converted to the frequency domain, and the updated method of the center frequency is obtained, where λ is updated at the same time. The specific expression is as follows:

(20) $$\;\;{\hat{u}}_{k}^{n+1}\left(\omega \right)=\frac{\hat{f}\left(\omega \right)-\sum_{i\neq k}{\hat{u}}_{i}\left(\omega \right)+\frac{\hat{\lambda }\left(\omega \right)}{2}\;\;}{1+2\alpha {\left(\omega -\omega_{k}\right)}^{2}},$$

(21) $$\;\;\;\;\;\;\;\;\;\;\;\;\;\;\;\;\;\;\;\;\;\;\;\;\;\;\;\;\;\;\;\;\;\;\;\;\;\;\;\;\;\;\;\;\;\;\omega_{k}^{n+1}=\frac{\int_{0}^{\infty }\omega {\left|{\hat{u}}_{k}\left(\omega \right)\right|}^{2}\;d\omega }{\int_{0}^{\infty }{\left|{\hat{u}}_{k}\left(\omega \right)\right|}^{2}\;d\omega }\;\;\;,$$

(22) $${\hat{\lambda }}^{n+1}\left(\omega \right)\leftarrow {\hat{\lambda }}^{n}\left(\omega \right)+\tau \left(\hat{f}\left(\omega \right)-\underset{k\;}{\sum }{\hat{u}}_{k}^{n+1}\left(\omega \right)\right),$$

On the whole, the frequency center and bandwidth of each IMF component are continuously updated in the process of iteratively solving the variational model until the iterative stop condition is satisfied, $\sum_{k}||{\hat{u}}_{k}^{n+1}-{\hat{u}}_{k}^{n}|{|}_{2}^{2}/||{\hat{u}}_{k}^{n}|{|}_{2}^{2}<e$. For a given discrimination accuracy *e* > 0, the whole cycle is ended, and finally, *K* narrowband IMF components are obtained according to the frequency domain characteristics of the actual signal, which completes the adaptive segmentation of the signal frequency band, effectively avoiding modal aliasing.

The Hilbert–Huang transform is described here. The VMD method can adaptively decompose the signal $u_{k}\left(t\right)$ into *K* IMFs. The instantaneous frequency has a concrete physical meaning, and the instantaneous frequency and instantaneous amplitude of each IMF can be calculated. The Hilbert transformation of each IMF in the Equation (22) is:

(23) $$H\left({\mathrm{IMF}}_{i}\left(t\right)\right)=\frac{1}{\pi }\int_{-\infty }^{+\infty }\frac{{\mathrm{IMF}}_{i}\left(\tau \right)}{t-\tau }d\tau .$$

The analytical signal structure of the IMF component is as follows

(24) $$z_{i}\left(t\right)={\mathrm{IMF}}_{i}\left(t\right)+jH({\mathrm{IMF}}_{i}\left(t\right))=a_{i}\left(t\right)e^{j\emptyset_{i}\left(t\right)}.$$

Calculate the amplitude function and phase function of the available IMF components:

(25) $$a_{i}\left(t\right)=\sqrt{{\mathrm{IMF}}_{i}^{2}\left(t\right)+H^{2}\left({\mathrm{IMF}}_{i}\left(t\right)\right)},\;\emptyset_{i}=arctan\frac{H\left({\mathrm{IMF}}_{i}\left(t\right)\right)}{{\mathrm{IMF}}_{i}\left(t\right)}$$

Differentiate the phase function to obtain the instantaneous frequency of the IMF component as follows

(26) $$f_{i}\left(t\right)=\frac{1}{2\pi }\omega_{i}\left(t\right)=\frac{1}{2\pi }\cdot \frac{d\emptyset_{i}\left(t\right)}{dt}.$$

The above formula is called the Hilbert amplitude spectrum of the signal $u_{k}\left(t\right)$ as:

(27) H(ω,t)=Re∑i=1Kai(t)ej∫ωi(t)dt.

The Hilbert marginal spectrum of the signal $u_{k}\left(t\right)$ is the time variable integral of the Hilbert spectrum, which is defined as follows:

(28) $$h\left(\omega \right)=\int_{0}^{T}H\left(\omega ,t\right)dt\;\;\;\;\;\;\;\;\;\;\;\;\;\;\;\;\;\;\;$$

In the above equation, *T* represents the length of the signal. The Hilbert amplitude spectrum of the signal can be used to comprehensively describe the changes of the signal with time and frequency in a three-dimensional graph composed of time, frequency and amplitude, while the Hilbert marginal spectrum reflects the law of the amplitude of the signal with frequency.

**Figure 1:**
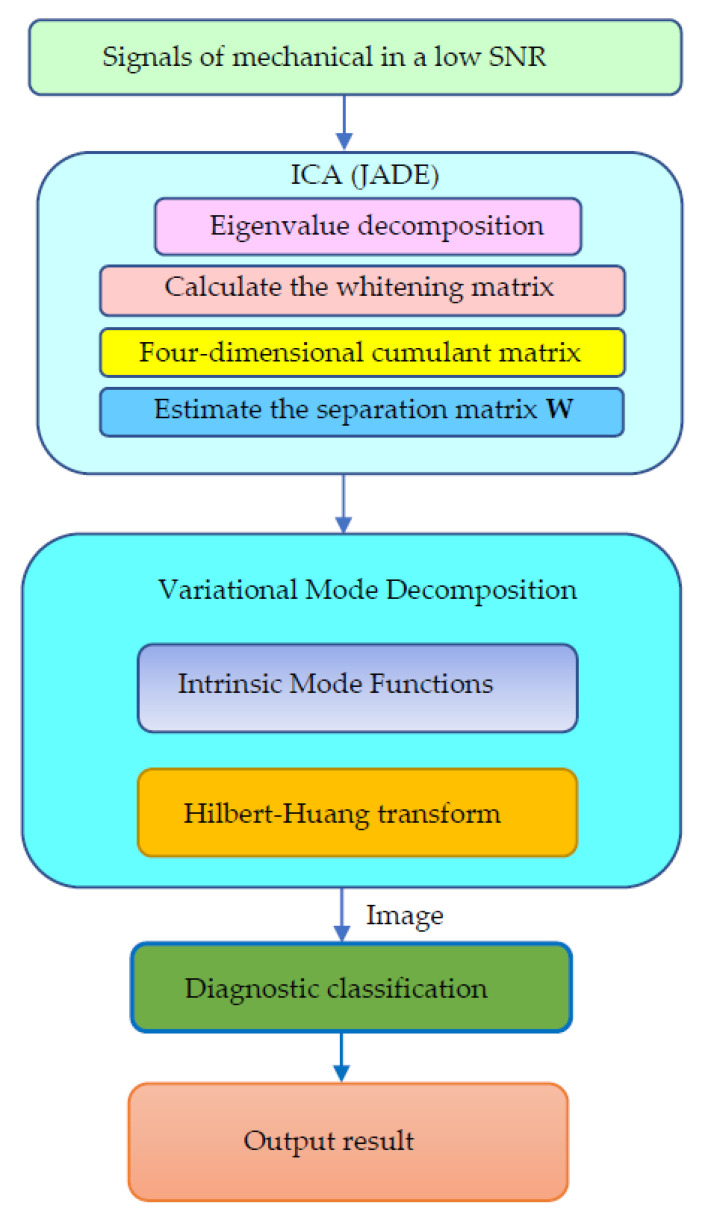
The entire flowchart of ICA-VMD.

Figure 1 provides the entire flow chart of ICA-VMD and explains how to implement it. There are mechanical systems that obtain signals through sensors at low SNR. JADE based on the above ICA theory separates noise and main components. The main component obtains the IMF through VMD, and then obtains the time-frequency diagram through Hilbert-Huang transform.

## 3. Results

The cocktail party problem is the most famous example of ICA applications [40]. There are five different positions—s_1_, s_2_, s_3_, s_4_, and s_5_, of which five original sources are contaminated by white Gaussian noise. In the simulation, s_1_(*t*), s_2_(*t*), s_3_(*t*), s_4_(*t*), and s_5_(*t*) are, respectively, 5, 10, 15, 20, and 25 Hz sine waveforms, and n(*t*) represents white Gaussian noise. When six sound sources appear at the same time, two sensors are installed in different positions to record sound. Only two sensors are used to measure six sources. The signals received by the two sensors can be expressed as:

x_1_(*t*) = a_11_s_1_(*t*) + a_12_s_2_(*t*) + a_13_s_3_(*t*) + a_14_s_4_(*t*) + a_15_s_5_(*t*) + n(*t*) x_2_(*t*) = a_21_s_1_(*t*) + a_22_s_2_(*t*) + a_23_s_3_(*t*) + a_24_s_4_(*t*) + a_25_s_5_(*t*) + n(*t*) (29)

where a*_ij_*(*i* = 1,2, *j* = 1,…,5) are different parameters, depending on the distance between the microphone and the speaker, and a*_ij_* represents the elements of the ***A*** matrix. In this case, the element of matrix ***A*** is randomly selected because ***A*** is unknown, and it is a parameter related to the distance from the sound source to the microphone. However, in order to obtain a low SNR, the noise parameter is increased. These are constants chosen at random. Let us take the following mixing matrix:

$$A=\left[\begin{array}{cc} 1 & 3\\ 2 & 4 \end{array}\;\;\;\;\begin{array}{c} 3\\ 2 \end{array}\;\;\;\;\begin{array}{ccc} 2 & 4 & 30\\ 1 & 3 & 30 \end{array}\right]$$

**Figure 2:**
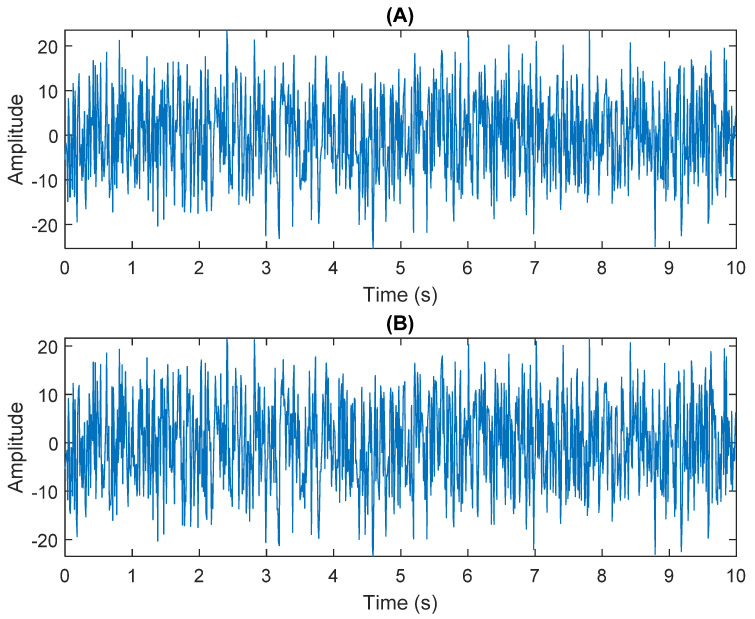
The numerical simulation of data received by two sensors: (**A**) In Equation (29) the sensor signal ×1. (**B**) In Equation (29) the sensor signal ×2.

**Figure 3:**
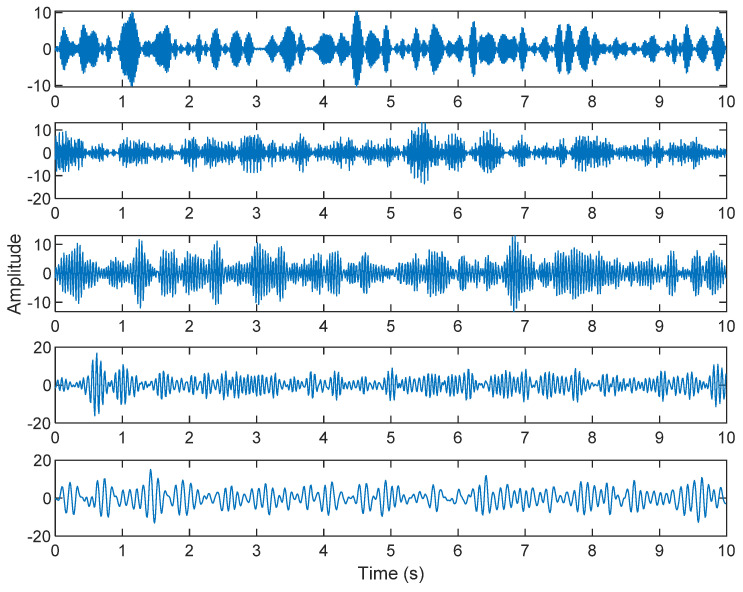
The results of the VMD component obtained from the numerical simulation of the sensor.

**Figure 4:**
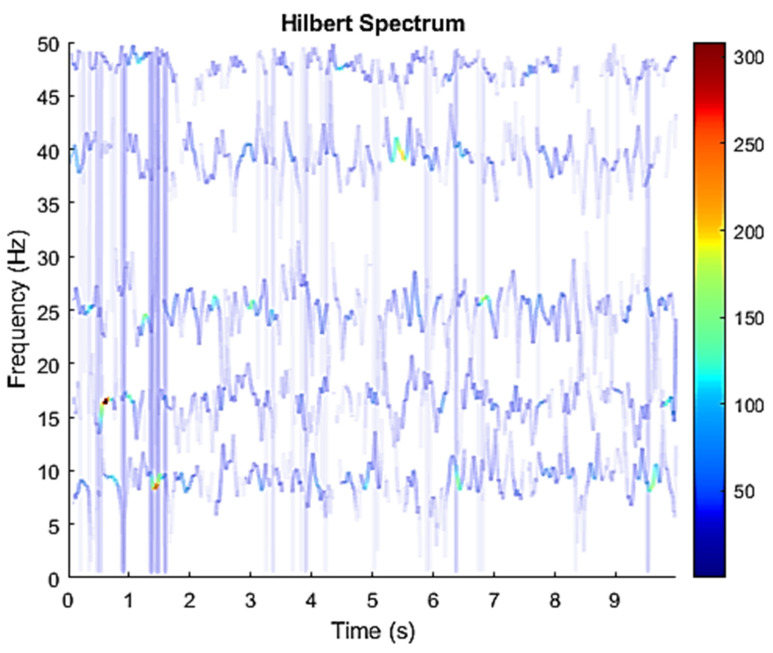
Hilbert Spectrum results of the VMD component obtained from the numerical simulation of the sensor.

The row parameters in the A matrix cannot be exactly the same. Otherwise, the two sensors receive the same signal and are dependent, and ICA will not work. The SNR of white Gaussian noise and the original signal is –6.46 dB. Figure 2 shows the time domain of the data received by the two sensors. Figure 3 shows the VMD component obtained by direct application of numerical simulation. VMD cannot separate the noise from the signal or decompose the signal into its constituent components under low SNR conditions. Figure 4 shows the results of the Hilbert spectrum of the VMD component obtained from the numerical simulation of the sensor. At this point, ICA finds the linear transformation ***W*** of the relevant sensor ***X***. The ***W*** matrix is expressed as follows:

$$W=\left[\begin{array}{cc} 0.02376 & -0.12907\\ 0.63200 & -0.62810 \end{array}\;\;\right]$$

**Figure 5:**
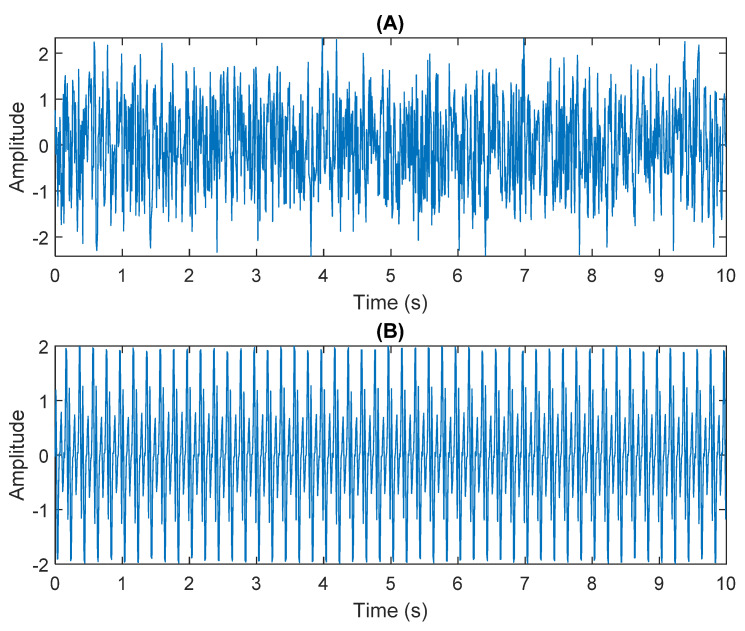
Analysis of data separated by ICA: (**A**) white Gaussian noise; (**B**) mixed sources.

**Figure 6:**
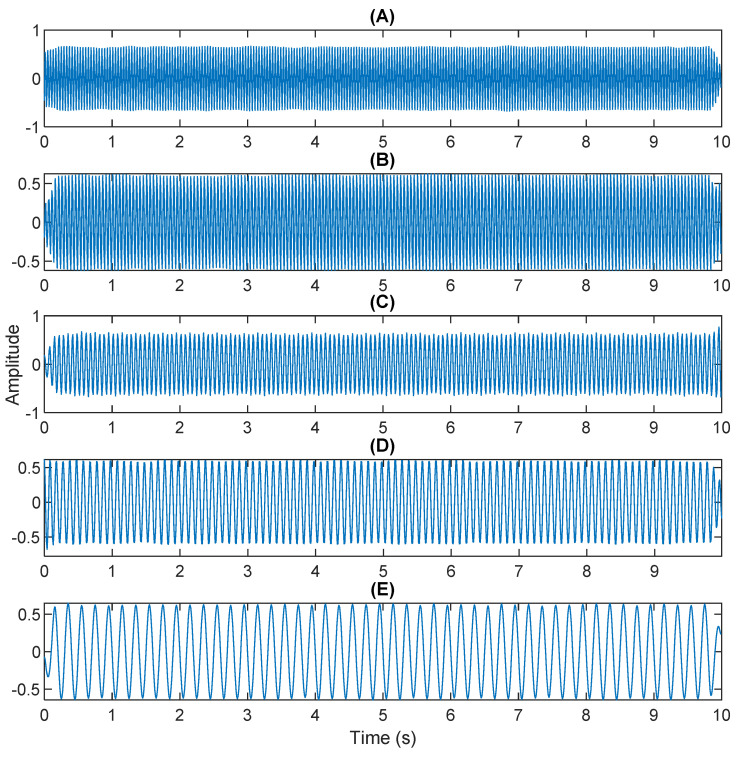
Analysis of data separated by ICA-VMD: (**A**) 25 Hz sin waveform; (**B**) 20 Hz sin waveform; (**C**) 15 Hz sin waveform; (**D**) 10 Hz sin waveform; (**E**) 5 Hz sin waveform.

**Figure 7:**
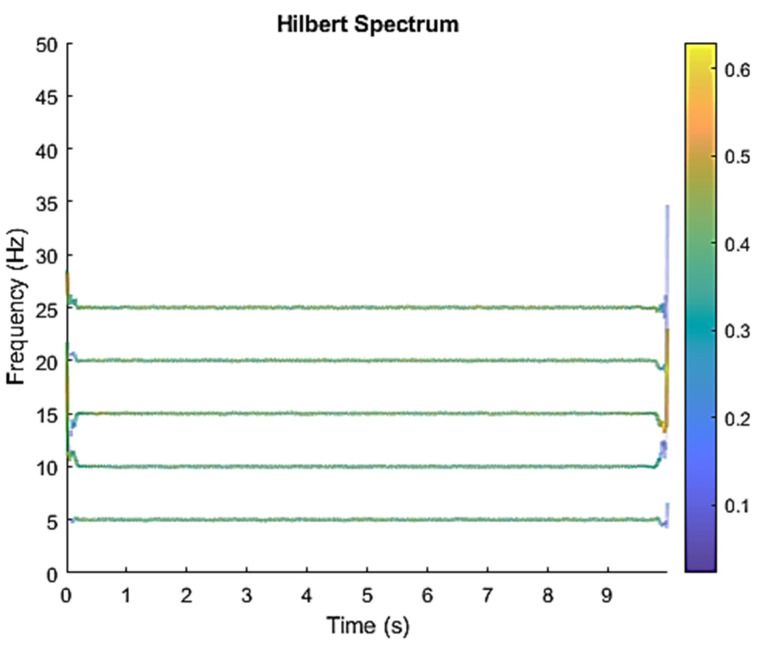
Analysis of data separated by ICA-VMD’s Hilbert Spectrum results.

**Figure 8:**
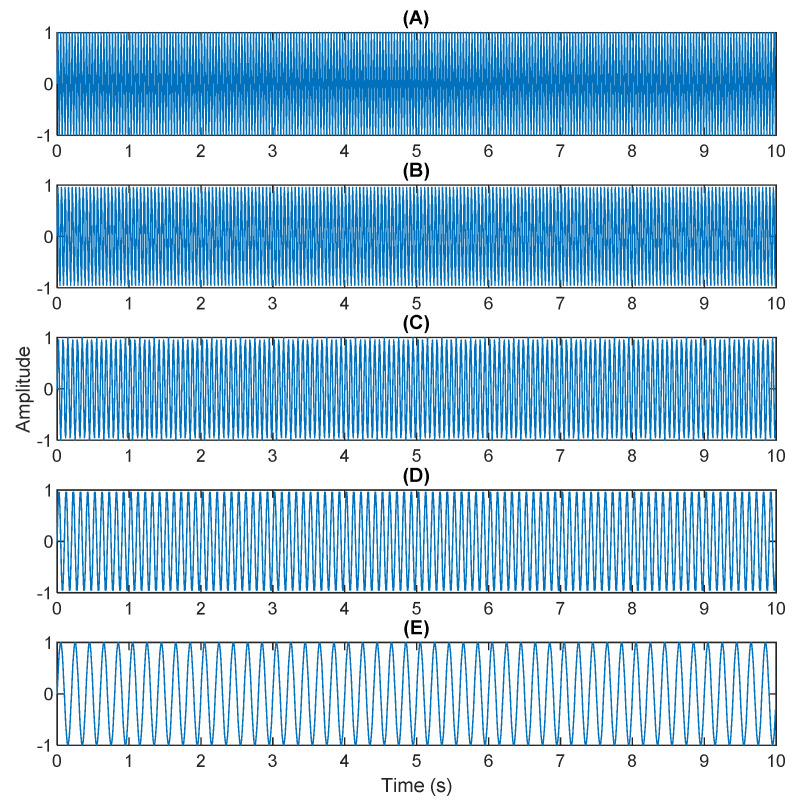
Original sources: (**A**) 25 Hz sin waveform; (**B**) 20 Hz sin waveform; (**C**) 15Hz sin waveform; (**D**) 10 Hz sin waveform; (**E**) 5 Hz sin waveform.

**Figure 9:**
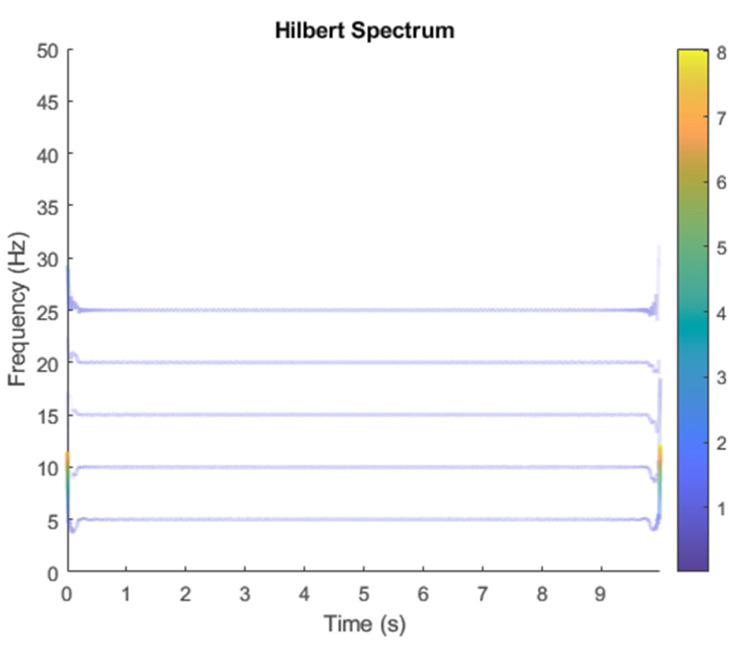
Analysis of original sources by Hilbert Spectrum results.

**Figure 10:**
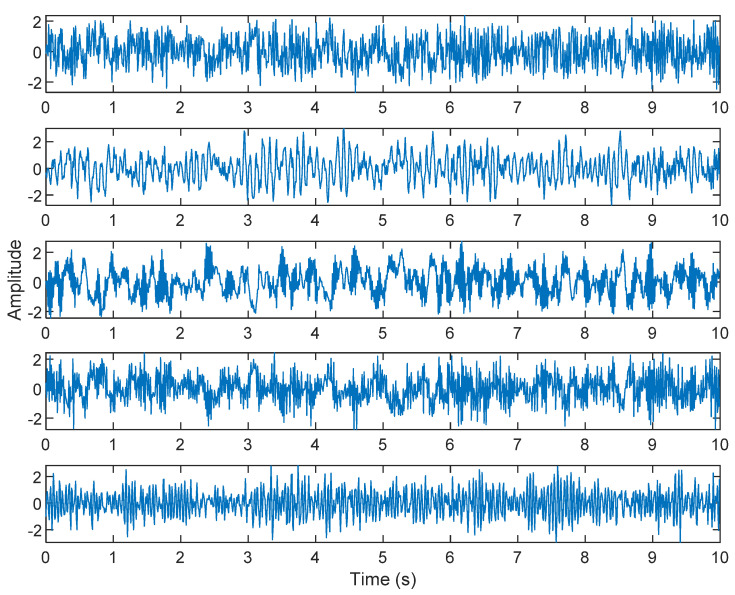
Analysis of data separated by VMD-ICA.

**Figure 11:**
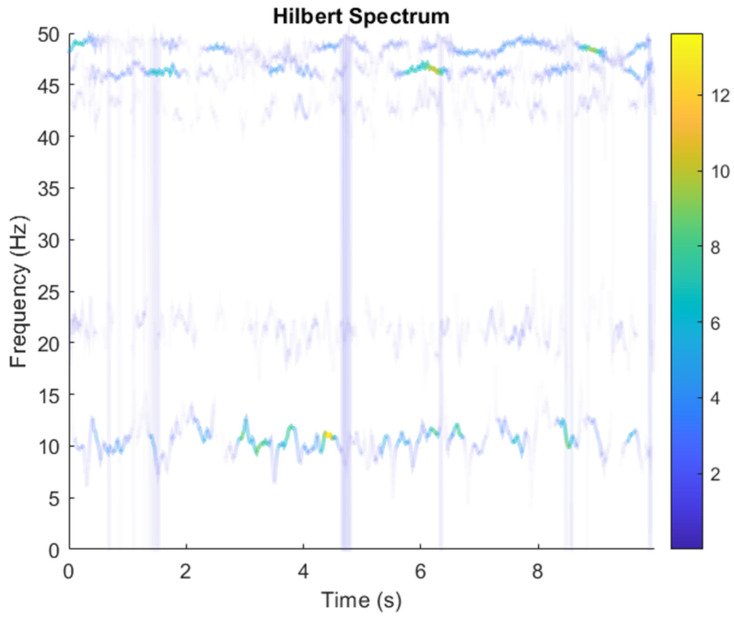
Analysis of data separated by VMD-ICA’s Hilbert Spectrum results.

The multiplication of matrices ***W*** and X, such as in Equation (3), provides the ICA output Y. The result of estimating Y through ICA can be seen in Figure 5. One is noise and the other comes from mixed sources. After successfully separating the signal and noise, the VMD method can be used to decompose the data from these mixed sources into its component parts. Figure 6 shows the ICA-VMD components obtained from the data. The VMD process reduces the analyzed time series into components, such as IMF, so as to “sift” or separate the different frequency scales of the data. The screening is undertaken adaptively, without imposing an a priori structure on the data. The screening first identifies and picks out the components with the highest frequencies, and then performs the same screening on the lower frequencies. Figure 7 shows the separated data of ICA-VMD’s Hilbert Spectrum results. Figure 7 shows the time–frequency diagram of the original data of 5, 10, 15, 20, and 25 Hz sine waveform. Looking at the results in detail, the waveform is distorted, and the results cannot be exactly the same as the original signal. This is due to two points. First, because ***W*** estimated by ICA is not the inverse matrix of real ***A***; ***W*** is not the only one, as there may be multiple ***W***. Second, VMD still has an end effect and a mode mixing problem. Figure 8 and Figure 9 show the original signals s_1_(*t*), s_2_(*t*), s_3_(*t*), s_4_(*t*), and s_5_(*t*), which are 5, 10, 15, 20, and 25 Hz sine waveforms, respectively. Figure 10 and Figure 11 show the results of doing VMD first and then ICA. VMD-ICA cannot effectively recover the original signal in a low SNR environment. 

Since the simulation in the above case is a pure sine waveform, two complex signals are simulated next, and each signal is sampled at 3 kHz for 1 s. There are two different positions, s_1_ and s_2_, and these two original sources are polluted by white Gaussian noise. In the simulation, the first s_1_(*t*) signal is a quadratic chirp, and the time instance defined in array *t* generates linear sweep samples displaying frequency increases from 300 to 1300 Hz during the measurement. The second s_2_(*t*) signal is the instantaneous frequency of the composite chirp and has a sinusoidal frequency. The instantaneous frequency change is between 0 and 1250 Hz. n(*t*) represents white Gaussian noise. When three signals appear at the same time, the two sensors are installed in different positions to measure. Only two sensors are used to measure three sources. The signals recorded by the two sensors constitute the weighted sum of the three signals. The signals received by the two sensors can be expressed as:

x_1_(*t*) = a_11_s_1_(*t*) + a_12_s_2_(*t*) + n(*t*) x_2_(*t*) = a_21_s_1_(*t*) + a_22_s_2_(*t*) + n(*t*) (30)

where a*_ij_*(*i* = 1,2, *j* = 1,…,3) are different parameters, depending on the distance between the sensor and the signal, and a*_ij_* represents the element of the ***A*** matrix.

$$A=\left[\;\;\;\;\begin{array}{ccc} 2 & 3 & 30\\ 1 & 4 & 30 \end{array}\right]$$

**Figure 12:**
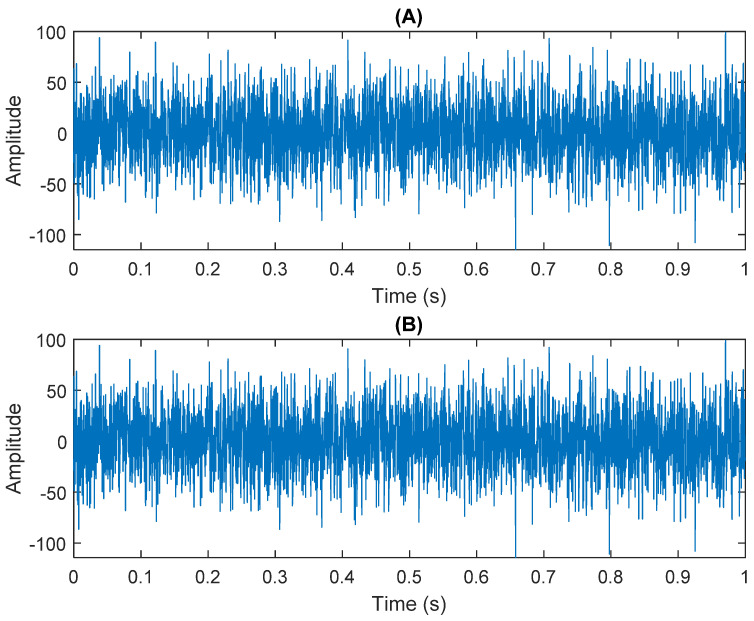
The numerical simulation of data received by two sensors: (**A**) In Equation (30) the sensor signal ×1. (**B**) In Equation (30) the sensor signal ×2.

**Figure 13:**
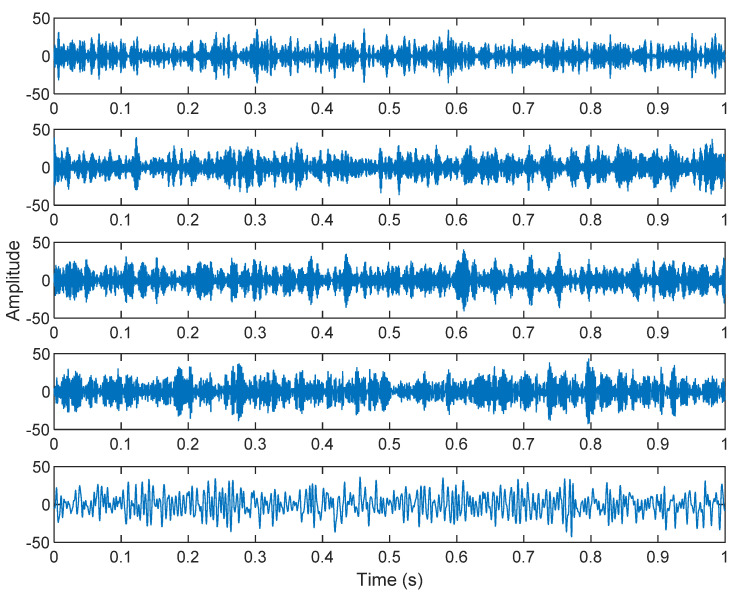
The results of the VMD component obtained from the numerical simulation of the sensor.

**Figure 14:**
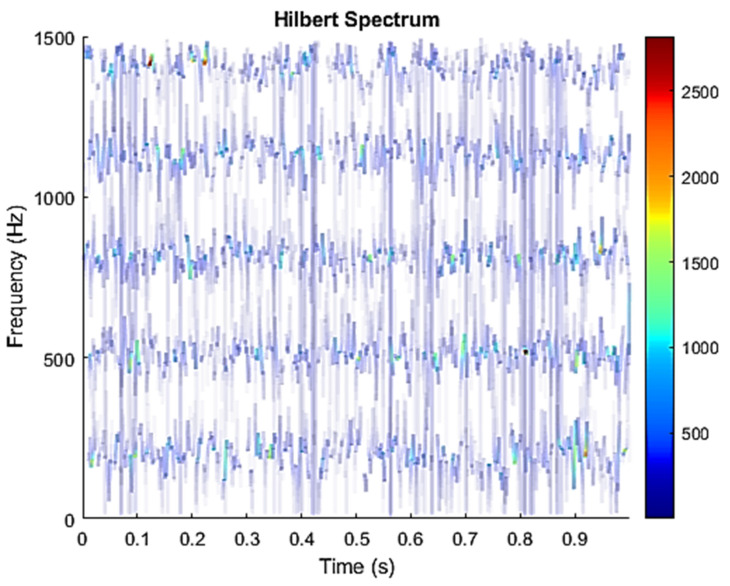
Hilbert Spectrum results of the VMD component obtained from the numerical simulation of the sensor.

In this research, the element of matrix ***A*** was randomly selected not because the element ***A*** is unknown in the real situation, but because the noise parameter is increased in order to obtain a low SNR. The SNR of white Gaussian noise and the original signal was –21.3728. Figure 12 shows the time domain of the data received by the two microphones. Figure 13 shows the VMD component obtained by direct application of numerical simulation. VMD cannot separate the noise from the signal or decompose the signal into its constituent components under low SNR conditions. Figure 14 shows the results of the Hilbert spectrum of the VMD component obtained from the numerical simulation of the sensor. Therefore, the goal of ICA theory is to obtain a separation matrix ***W*** through ***X***, so that the signal ***Y*** obtained by ***W*** acting on ***X*** is the optimal approximation of the independent source ***S***. The ***W*** matrix is expressed as follows:

$$W=\left[\begin{array}{cc} 0.07169 & -0.03842\\ 0.98836 & -0.98893 \end{array}\;\;\right]$$

**Figure 15:**
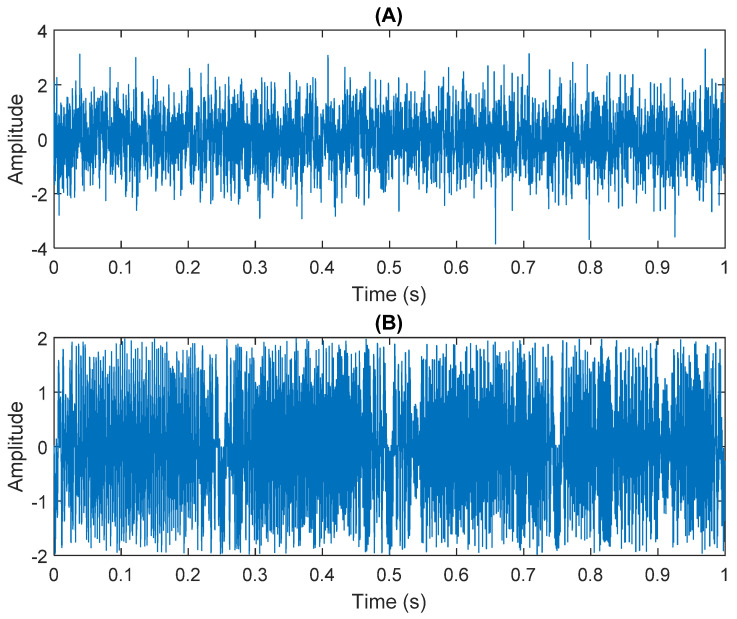
Analysis of data separated by ICA: (**A**) white Gaussian Gaussian noise; (**B**) mixed sources.

**Figure 16:**
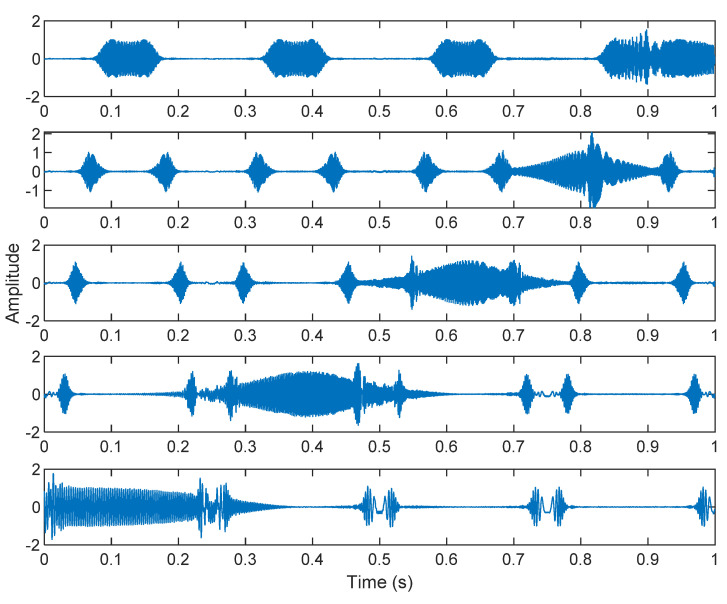
Analysis of data separated by ICA-VMD.

**Figure 17:**
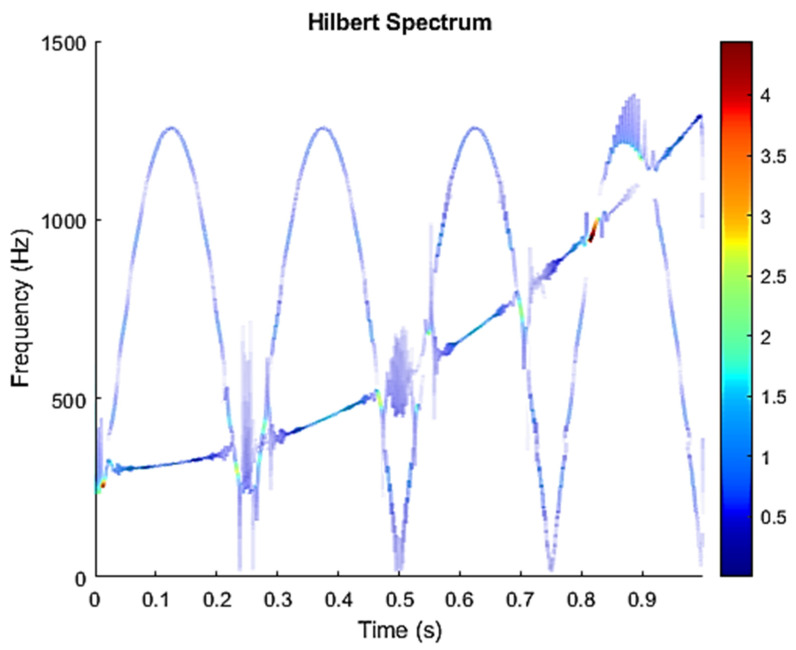
Analysis of data separated by ICA-VMD’s Hilbert Spectrum results.

**Figure 18:**
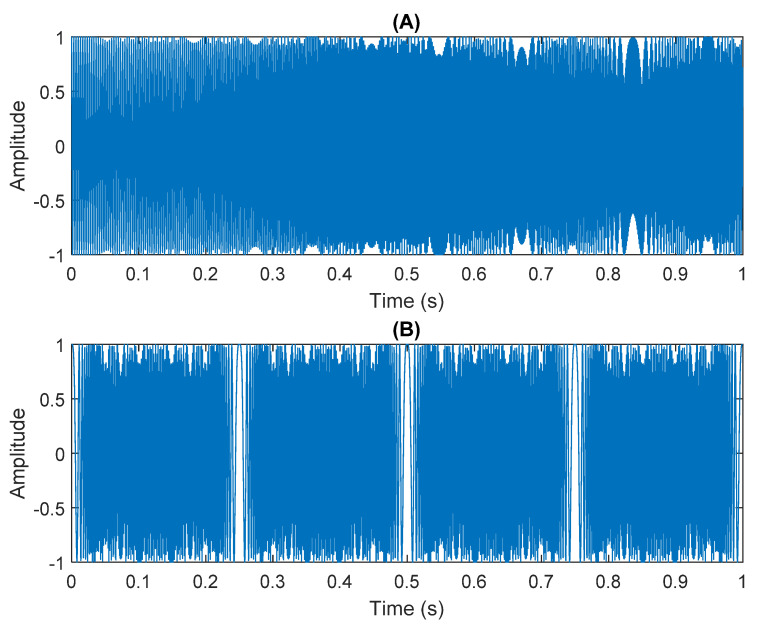
Original sources: (**A**) Quadratic chirp, the frequency of which increased from 300 to 1300 Hz during the measurement. (**B**) The instantaneous frequency of the compound chirp and the chirp with sinusoidally varying frequency content.

**Figure 19:**
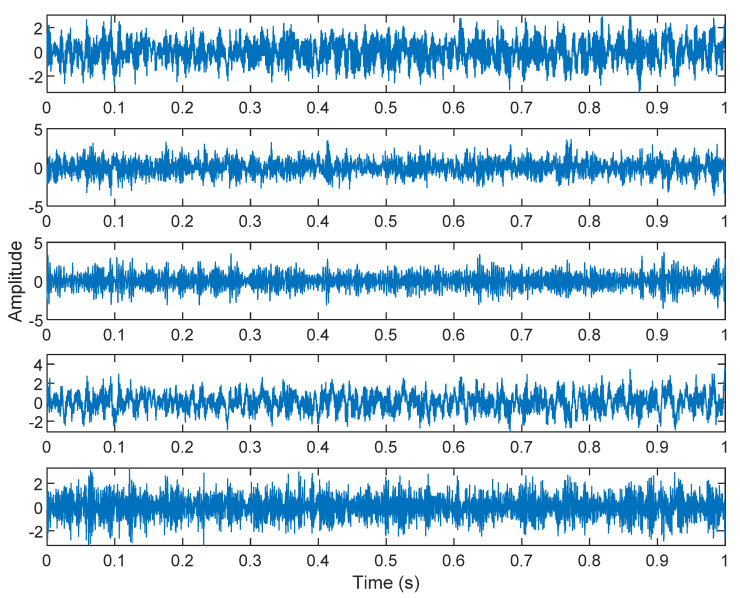
Analysis of data separated by VMD-ICA.

**Figure 20:**
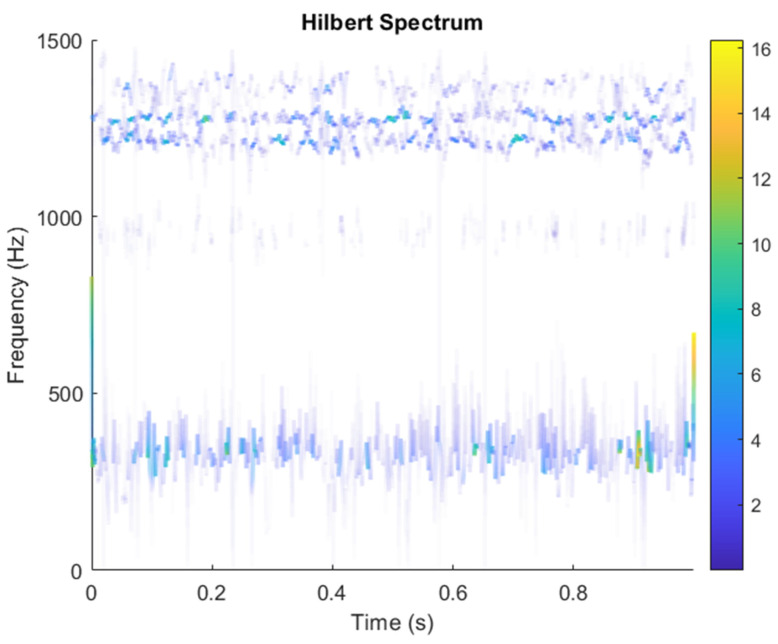
Analysis of data separated by VMD-ICA’s Hilbert Spectrum results.

In the ICA model, not only can the source signal not be directly obtained by the sensor, but also the coefficient matrix ***A*** cannot be obtained, that is, the combination of each source signal is also unknown in practice. Some necessary conditions must be set to ensure that the ICA model can be estimated and solved. The result of estimating Y through ICA can be seen in Figure 15. One is noise and the other comes from mixed sources. After successfully separating the signal and noise, the VMD method can be used to decompose the data from these mixed sources into their component parts. Figure 16 shows the ICA-VMD components obtained from the data. Figure 13 shows the separated data of ICA-VMD’s Hilbert Spectrum results. Figure 17 shows the time–frequency diagram of the original data s_1_(*t*) and s_2_(*t*). VMD must select several ***K*** parameters of IMF, as well as a small ***K*** to distort two complex signals, so five IMFs were selected here. Figure 18 shows that the two complex signals interfere with overlapping frequencies. This is due to the inherent phenomenon of VMD. Figure 19 and Figure 20 show the results of doing VMD first and then ICA. VMD-ICA cannot effectively recover the original signal in a low SNR environment.

In the third case, Case Western Reserve University (https://engineering.case.edu/bearingdatacenter/download-data-file (8 November 2021) was used to provide test data on faulty bearings of motors. Experiments were carried out using a one horsepower motor, and acceleration data were measured near and far from the motor bearing. Electrical discharge machining (EDM) was used to diagnose faults in motor bearings. A fault with a diameter of 0.021 inches (0.5334 mm) was introduced into the inner raceway. The faulty bearing was reinstalled into the test motor and the vibration data of the motor load of one horsepower (motor speed is 1774 RPM) were recorded. SKF bearings were used. Vibration data were collected using an accelerometer, which was connected to a housing with a magnetic base. The accelerometer was placed at the 12 o’clock position of the drive end and the fan end of the motor housing. A 16-channel DAT recorder was used to collect the vibration signal, and post-processing took place in the MATLAB environment. Then, 48,000 samples per second are collected for drive end bearing failures, and a total of 10 s was recorded. In the simulation, s_1_(*t*) was the fault data of the motor bearing and n(*t*) was the white Gaussian noise, which simulated the noise of other interferences. Two accelerometers were installed in different positions for the measurement. The signals received by the two accelerometers can be expressed as:

x_1_(*t*) = a_11_s_1_(*t*) + n(*t*) x_2_(*t*) = a_21_s_1_(*t*) + n(*t*) (31)

where a*_ij_*(*i* = 1,2, *j* = 1,…,2) are the different parameters, depending on the distance between the sensor and the signal, and a*_ij_* represents the element of the ***A*** matrix. Because the ***A*** element is unknown in the real situation, the matrix ***A*** element was randomly selected in this research, but the noise parameter was increased in order to obtain a low SNR. These were randomly selected constants using the following mixing matrix:

$$\;A=\left[\begin{array}{cc} 2 & 30\\ 3 & 30 \end{array}\;\;\;\;\right]$$

**Figure 21:**
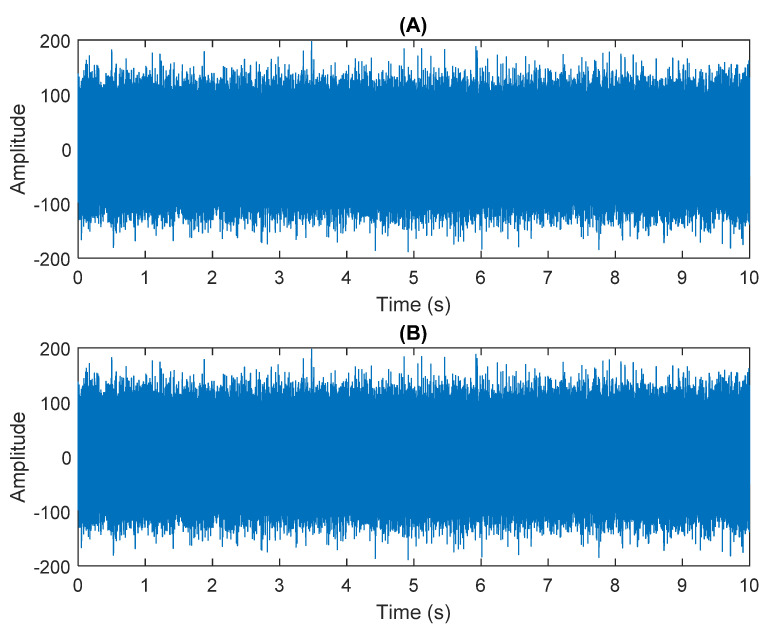
The numerical simulation of data received by two sensors: (**A**) In Equation (31) the sensor signal x1. (**B**) In Equation (31) the sensor signal x2.

**Figure 22:**
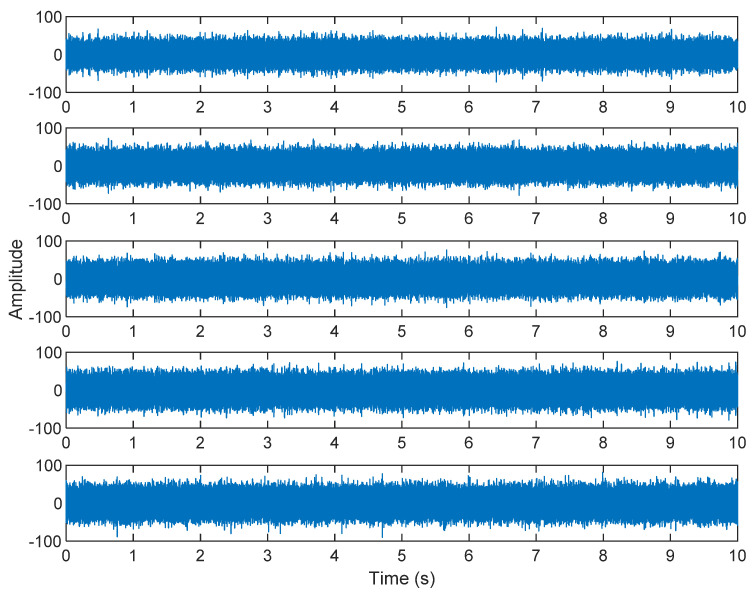
The results of the VMD component obtained from the numerical simulation of the sensor.

**Figure 23:**
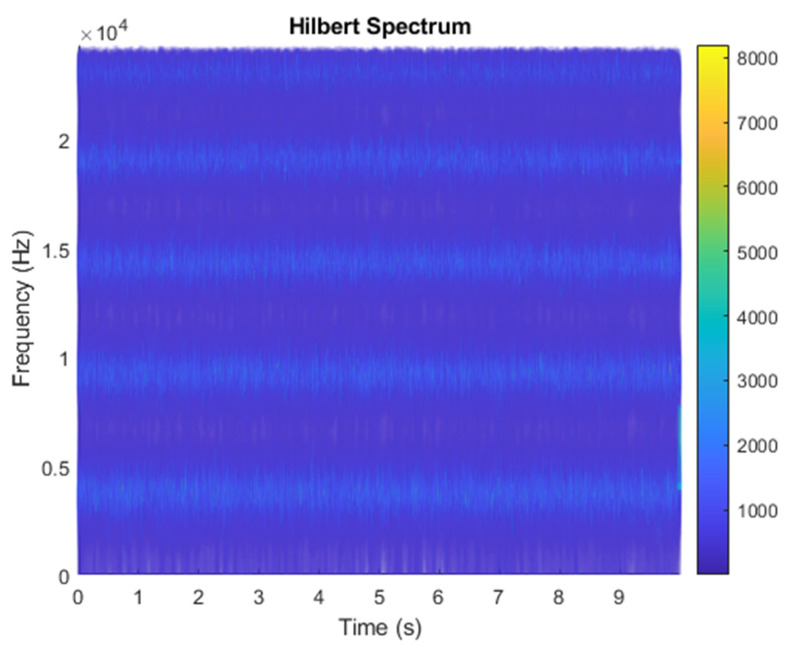
Hilbert Spectrum results of the VMD component obtained from the numerical simulation of the sensor.

However, the mixing matrix ***A*** is unknown in research. The SNR of white Gaussian noise and the original signal was –46.8177. Figure 21 shows the time domain of the data received by the two sensors. Figure 22 shows the VMD component obtained by direct application of numerical simulation. VMD cannot separate the noise from the signal or decompose the signal into its constituent components under low SNR conditions. Figure 23 shows the results of the Hilbert spectrum of the VMD component obtained from the numerical simulation of the sensor. The key of ICA is to find an optimal separation matrix ***W***, and then recover the source signal from the observed signal through a linear transformation. The ***W*** matrix is expressed as follows:

$$W=\left[\begin{array}{cc} 0.04950 & -0.02590\\ 10.91299 & -10.91293 \end{array}\;\;\right]$$

**Figure 24:**
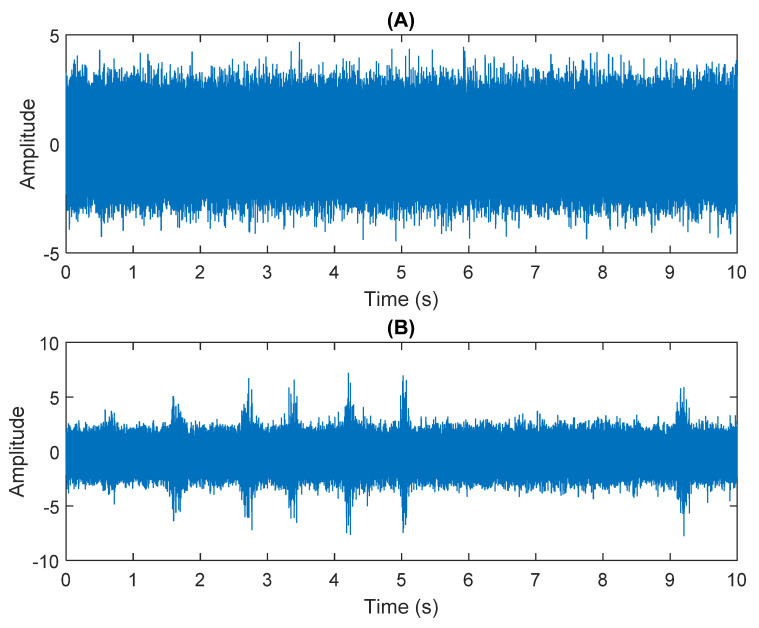
Analysis of data separated by ICA: (**A**) white Gaussian noise; (**B**) mixed sources.

**Figure 25:**
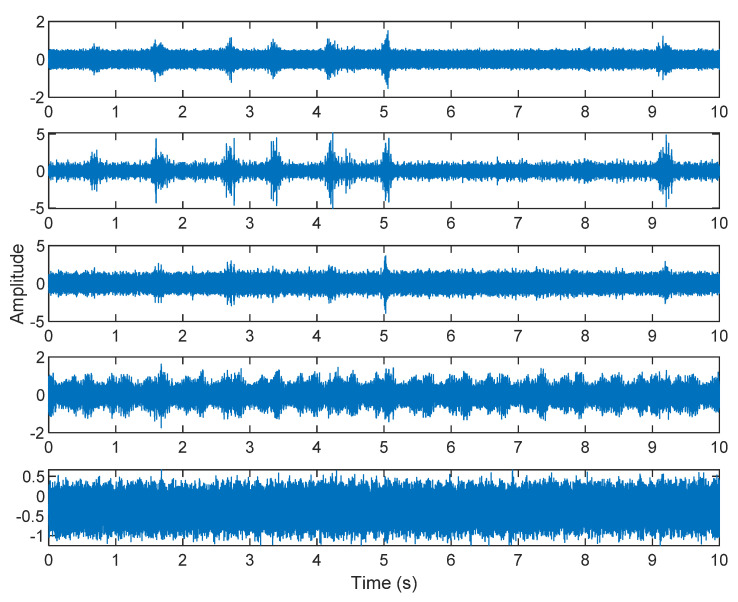
Analysis of data separated by ICA-VMD.

**Figure 26:**
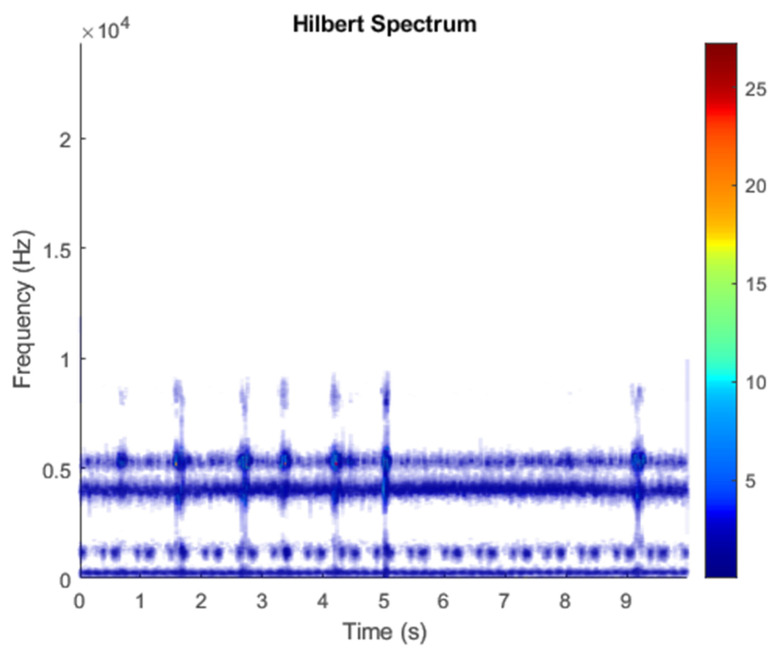
Analysis of data separated by ICA-VMD’s Hilbert Spectrum results.

**Figure 27:**
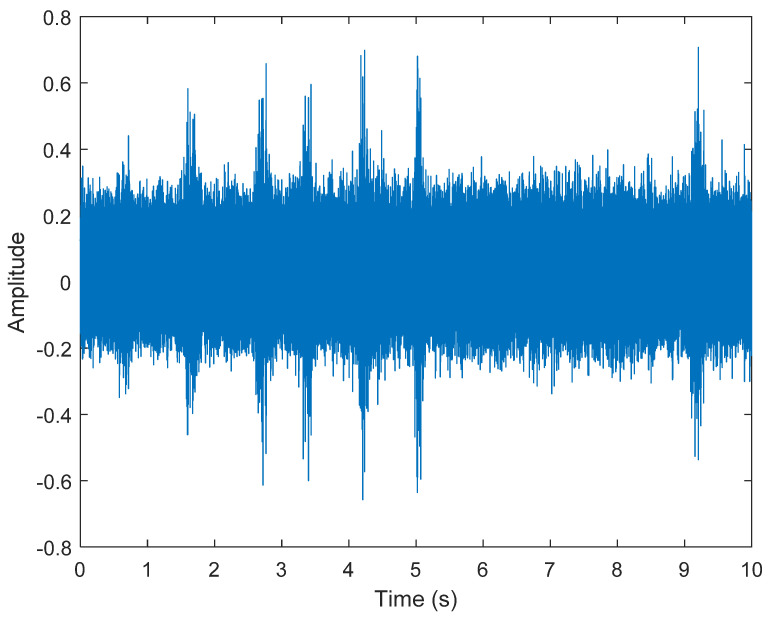
Original source of motor bearing failure.

**Figure 28:**
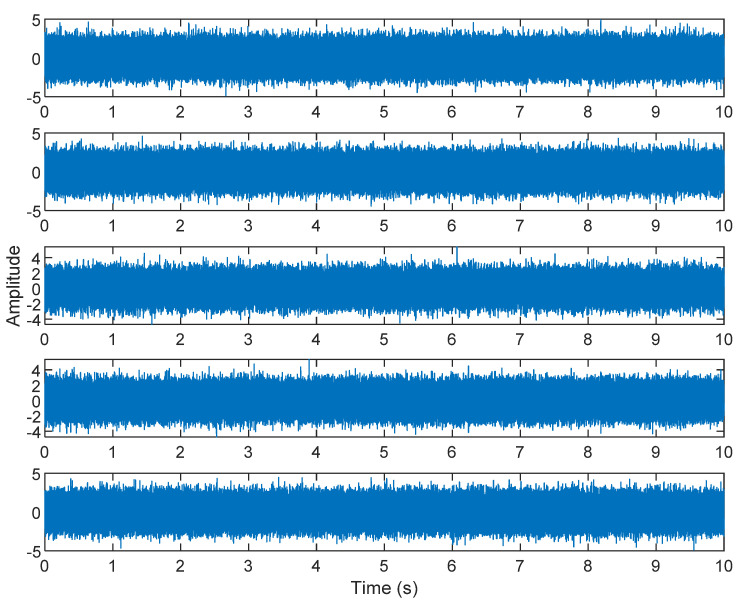
Analysis of data separated by VMD-ICA.

**Figure 29:**
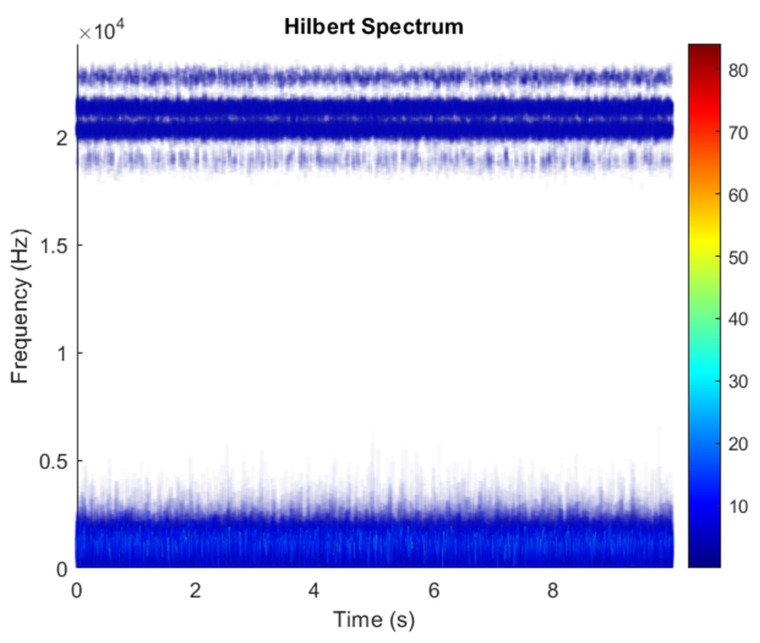
Analysis of data separated by VMD-ICA’s Hilbert Spectrum results.

After ICA finds the matrix ***W***, the result of multiplying ***X*** to estimate ***Y*** as in Equation (3) can be shown in Figure 24. ICA obtains two signals: one is noise and the other comes from a mixed source. After separating the signal and noise, the VMD method is used to decompose the data from these mixed sources into its component parts. Figure 25 shows the ICA-VMD components obtained from the data. Figure 26 shows the separated data of ICA-VMD’s Hilbert Spectrum results. Figure 27 shows the original source of motor bearing failure. The results show that ICA-VMD guarantees the effective restoration of the original data. This result can be clearly seen in 350, 1.3k, 4k, and 5.3k Hz, and blue is the frequency of the bearing rotation. The bearing failure points were at 0.6, 1.6, 2.8, 3.3, 4, 5, and 9 s, where the blue is more obvious and also shows a wider frequency. Figure 28 and Figure 29 show the results of doing VMD first and then ICA. VMD-ICA cannot effectively recover the original signal in a low SNR environment.

Next, explain the application conditions of ICA. In order to have a definite solution to the ICA problem, the following conditions must be met [17,18]:

(1) Each source signal is a real random signal with zero mean, and is statistically independent at any time.

(2) The number of observation signals m is greater than or equal to the number of source signals n, and the mixing matrix is a practically achievable matrix. When m > n, n observation signals are arbitrarily selected. At this time, the mixing matrix A is full rank and the inverse matrix exists.

(3) Only one Gaussian signal is allowed in the source signal, because two statistically independent Gaussian signals are still Gaussian signals after being mixed. Irrelevance, that is, always meets the independence requirements, so if there is more than one Gaussian signal in the source signal, the source signal is inseparable.

## 4. Conclusions

ICA and VMD each have their advantages and limitations. In this research contribution, the ICA-EMD method that combines the advantages of the two is proposed to achieve the complementary characteristics to maximize the performance. The low signal-to-noise ratio data that cannot be processed by traditional methods can now be analyzed using the combined ICA-VMD method under the ICA constraints and conditions of the two sensors. The pre-processing of ICA can separate one from noise and the other from mixed sources. After successfully separating the signal and noise, the VMD method can be used to decompose the data from these mixed sources into its component parts. There are three cases in the research results. These original sources were first contaminated by white Gaussian noise under different SNR conditions. The SNR of the first case is –6.46 dB, the SNR of the second case is –21.3728, and the SNR of the third case is –46.8177. ICA-VMD can effectively recover the original signal in the same case. Noise is inevitable in the measurement of research, and it is everywhere. Noise separation or reduction is a challenging area of signal processing data analysis. Vibration signals collected in the industrial field usually have a low signal-to-noise ratio, which is not sufficient to identify faults. Some noise in the research may be harmless, and some noise may be critical to the success or failure of the project. However, the disadvantage of ICA is that the number of sensors cannot be less than the source signal, otherwise some source signals cannot be completely separated. Both ICA and VMD are powerful methods, but they also have their limitations. The research contribution of this paper proposes an ICA-EMD method that combines the advantages of the two to improve their original limitations, in order to achieve the complementarity of characteristics to maximize performance. A balance between noise performance and computational complexity can be discussed in future research, and the performance of more noise removal algorithms can be compared with this research.

## Data Availability

Not applicable.
